# Is empathy-driven teaching effective in cultural product design? An empirical study on students’ empathy and design creativity

**DOI:** 10.3389/fpsyg.2026.1926260

**Published:** 2026-09-29

**Authors:** Mingzhu Yu, Xuan Qin

**Affiliations:** School of Art and Design, Ma’anshan University, Ma’anshan, Anhui, China

**Keywords:** cultural product design, design creativity, design education, design empathy, empathy-driven instructional approach

## Abstract

**Introduction:**

Cultural product design (CPD) education concerns not only students’ extraction and translation of traditional cultural elements, but also their understanding of the cultural contexts behind cultural artefacts and the experiences of potential users. Empathy is considered to help designers understand needs, emotions, and contexts from the perspectives of others; however, its role in CPD education remains insufficiently examined through empirical research. This study introduced an empathy-driven instructional approach to examine its associations with students’ empathy and design creativity.

**Methods:**

The participants were 79 undergraduate students from two intact classes in a CPD course. The two classes were assigned at the class level to either the empathy-driven instructional approach or the traditional instructional approach. The empathy-driven group received systematic guidance based on the empathy-driven instructional approach, whereas the traditional group received the traditional instructional approach. Both groups completed design tasks based on designated cultural artefacts.

**Results:**

The results showed that students in the empathy-driven group demonstrated higher levels of empathy than those in the traditional group. The analyses also identified significant indirect associations through empathy between the instructional approach and the novelty and quality of design outcomes, a pattern consistent with partial mediation.

**Discussion:**

These findings suggest that the empathy-driven instructional approach in CPD education can help students develop a more integrated understanding of the relationships among artefact contexts, user experiences, and contemporary product experiences, and may support the generation of design outcomes with greater novelty and quality. This study provides preliminary empirical evidence for incorporating empathy training into CPD education.

## 1 Introduction

In contemporary design discourse, culture and design have become increasingly intertwined. Culture not only offers abundant referential resources for design, but also provides a critical foundation for design differentiation and identity expression (Lin, 2007; Lin et al., 2007; Moalosi et al., 2010; Chai et al., 2015). Among various cultural resources, ancient artefacts are frequently regarded as an inexhaustible source of inspiration (Wang and Ajovalasit, 2020; Dong et al., 2023; Qin et al., 2025). By drawing on artefacts’ forms, motifs, and colour schemes, designers attempt to establish formal connections between tradition and modernity. However, prior research suggests that the value of cultural products is not confined to function and form; rather, it is fundamentally rooted in the symbolic meanings and social emotions they embody (Hsu et al., 2011; Wang and Ajovalasit, 2020). Consumers use culturally evocative products to articulate identity, belonging, and affective attachment (Van Rompay, 2008). In essence, cultural products are not merely material objects, but media through which culture and emotion are communicated and negotiated (Ya-Juan Gao et al., 2018; Gumulya et al., 2023).

Within design education, this understanding raises a further challenge: when students are confronted with a large corpus of ancient artefacts, how can they create cultural products that are both creatively compelling and capable of eliciting emotional resonance? In response, existing studies have explored multiple approaches and perspectives (Luo and Dong, 2017; Qin et al., 2025). For example, Dong et al. (2023) developed cultural guidelines to help students interpret the cultural meanings embedded in artefacts, thereby enhancing students’ design creativity (Dong et al., 2023). Wang and Ajovalasit (2020) proposed a culturally sensitive design toolkit based on cultural feature categorization and mappings to emotional responses, aiming to guide designers in the early ideation phase to better align cultural characteristics with users’ emotional experiences (Wang and Ajovalasit, 2020).

Although the aforementioned studies provide useful support for interpreting cultural information and cultural meanings, they are largely delivered in predetermined formats. As a result, students are often positioned as passive recipients in most cases. Moreover, limited attention has been paid to whether students genuinely enter the cultural contexts in which artefacts are situated, and there is insufficient guidance to help them consider, from the users’ perspective, how cultural content may elicit emotional resonance in real-world use.

To address these limitations, it is necessary to incorporate instructional support oriented toward the development of empathy within course design. Empathy is widely regarded as a point of departure in the design thinking process. Its central function is to orient designers to understand problems from others’ perspectives, thereby enabling a deeper appreciation of others’ emotions, needs, and circumstances (Kouprie and Visser, 2009; Tracey and Baaki, 2022; Chen G. et al., 2025; Chen J. et al., 2025).

Accordingly, this study employs an empathy-driven instructional approach to guide students to attend to both the cultural contexts embedded in artefacts and users’ emotional experiences throughout the CPD process. The study focuses on (1) how the empathy-driven instructional approach is operationalized within the course, and (2) how changes in students’ empathy during the design process are reflected in their design outcomes. Based on this rationale, the following research questions (RQs) are proposed:

*RQ1*: In the context of CPD, can an empathy-driven instructional approach enhance design students’ empathy?

*RQ2*: Does an empathy-driven instructional approach improve students’ design creativity in CPD?

*RQ3*: Does an increase in empathy influence students’ design creativity in CPD?

## 2 Literature review

### 2.1 Research related to CPD

CPD typically refers to design activities that integrate traditional cultural meanings with contemporary lifestyles, technological conditions, and functional requirements, thereby creating cultural significance and emotional value while fulfilling utilitarian functions (Hsiao and Chou, 2004). Compared with general product design, CPD places greater emphasis on cultural connotations, identity construction, and value creation. It treats culture as a pivotal resource for shaping a product’s core value and differentiated competitiveness, enhancing product recognizability through cultural symbols and narratives and eliciting users’ emotional resonance (Wang and Ajovalasit, 2020).

Within this context, prior studies have proposed a range of process models that aim to systematize the transformation of culture into design (Moalosi et al., 2010; Gumulya et al., 2023). Representative of these, Lin (2007) proposed a CPD process that organizes design activities into three stages: identification, translation, and implementation. In this process, designers first identify relevant cultural elements, then translate them into design language and creative concepts, and finally materialize them as specific product features through iterative evaluation (Lin, 2007; Lin et al., 2007). Hsu et al. (2011) proposed a four-stage CPD framework comprising “identification-investigation-interaction-implementation” (Hsu et al., 2011). From the perspective of cultural enculturation, Gao et al. (2018) advanced a CPD framework grounded in human-culture interaction, arguing that the continuity of traditional culture in contemporary products can be achieved through contextualized and interactive design (Gao et al., 2018).

In studies of CPD education, existing literature has mainly focused on the cognition and application of cultural elements, the pedagogical role of design guidelines, and the effects of different types of cultural inspiration on creativity. Relevant studies have shown that students are generally more likely to identify and apply explicit cultural elements, such as form, colour, and patterns, but often show limitations in understanding and transforming deeper cultural elements at the behavioural and spiritual levels (Yang et al., 2022). Design-guideline-based analytical frameworks can help students identify and translate cultural resources more systematically and improve the novelty and quality of design solutions, although they may also lead to convergent ideation paths or design fixation (Dong et al., 2023). In addition, the level of abstraction of cultural inspiration can influence students’ cognitive load, associative pathways, and creative performance. More abstract cultural inspirations may better stimulate flexibility and originality, but they also impose greater demands on understanding and transformation (Qin et al., 2025).

Overall, existing research has remained largely focused on cognitive processing and methodological guidance, while paying less attention to designers’ own affective experiences, cultural identities and standpoints, and to how they may better understand the relationship between ancient cultural artefacts and contemporary users.

### 2.2 Empathy: conceptual dimensions and its role in design

Empathy refers to an individual’s capacity to understand others’ emotional experiences and psychological states from their perspectives through cognitive processing (Spreng et al., 2009). Empathy is commonly conceptualized as comprising two core dimensions, namely affective empathy and cognitive empathy (Harley et al., 2020; Li et al., 2024a). Affective empathy is primarily manifested as an immediate emotional response to others’ affective states (Spreng et al., 2009), with typical forms including emotional resonance or emotional contagion, for example, experiencing a similar emotion when observing another person’s joyful expression. Cognitive empathy emphasizes the ability to understand others’ emotions and circumstances through perspective taking. Its core lies in temporarily adopting another person’s standpoint at the cognitive level and grasping their emotions, behaviours, and ways of interpreting a situation (Baron-Cohen and Wheelwright, 2004).

Because design activities inherently involve understanding others’ needs, contexts, and experiences, empathy has increasingly been incorporated into design research. It is widely regarded as a point of departure for design thinking and one of the core elements of user-centered design (Kouprie and Visser, 2009; Tracey and Baaki, 2022; Drouet et al., 2024). Through empathy, designers are able to immerse themselves in users’ lived contexts, thereby identifying and framing design problems and underlying needs with greater accuracy. Empathy also provides an essential foundation for the stimulation of creative thinking (Chang et al., 2022; Chen G. et al., 2025; Chen J. et al., 2025; Shi et al., 2025). Empirical evidence further indicates a positive association between empathy and creative performance (Genco et al., 2011; Johnson et al., 2014; Lee and Park, 2021; Chang et al., 2022; Li et al., 2024a). In addition, studies suggest that, within design education contexts, educational interventions can effectively enhance students’ subjective perceptions of their own empathy (Ward, 2016; Archer and Turner, 2023).

In recent years, empathy has increasingly been introduced into design education and has evolved from a general orientation toward user understanding into an operational pedagogical strategy. Existing studies have mainly used role-playing, experiential prototyping, empathy maps, user feedback, and reflective activities to guide students into others’ situations, identify user needs, and continuously revise their own design judgements during the design process. For example, Shi et al. (2025) guided design students to understand the mobility difficulties and emotional experiences of specific users in real-life contexts by simulating the public transport experience of people with mild visual impairment (Shi et al., 2025). Chen G. et al. (2025); Chen J. et al. (2025) introduced user feedback at the early stage of a design project, prompting students to reflect on their judgements of user needs and further enhancing cognitive empathy, empathic motivation, and design creativity (Chen G. et al., 2025; Chen J. et al., 2025).

Accordingly, empathy in design education can be understood as a situated and reflective design capability. Through perspective-taking, contextual understanding, and emotional response, students are able to understand others’ experiences and develop more appropriate design judgements. However, existing research has mainly focused on accessible design, service design, learning experience design, or general design projects, with limited attention to how empathy can be applied in CPD education. In fact, CPD is highly dependent on designers’ understanding of cultural subjects, cultural contexts, and cultural sensitivity (Wang and Ajovalasit, 2020). It is therefore necessary to introduce an empathy-driven instructional approach into CPD education, guiding students to move beyond the identification and translation of cultural elements and to pay greater attention to historical users, contemporary users, and their emotional experiences.

Based on this, the present study defines empathy in the context of CPD as the designer’s ability, during the translation of cultural artefact elements, to understand the use contexts, user needs, emotional experiences, and cultural meanings of artefacts from the dual perspectives of historical and contemporary users, and to interpret the relationship between artefact meanings and contemporary product experiences from these dual user perspectives. Based on previous evidence that instructional interventions can foster the development of empathy, the following hypothesis is proposed:

*H1*: Compared with the traditional instructional approach, the empathy-driven instructional approach will be associated with a greater improvement in students’ empathy during the CPD process.

### 2.3 Theoretical frameworks of empathy in design contexts

Empathy is a dynamically unfolding process, and scholars have proposed multiple design empathy frameworks from a process perspective (Smeenk et al., 2019; Tracey and Baaki, 2022). For example, Hess and Fila (2016a, 2016b) developed a four-dimensional theoretical framework: (1) imagine-other perspective-taking, which refers to designers imagining how users might think and feel; (2) empathic concern, which refers to designers’ care and compassion for users’ circumstances; (3) emotional congruence, which refers to designers experiencing the same or similar emotional states as users; and (4) imagine-self perspective-taking or projection, namely designers imagining how they themselves would think and feel if placed in the users’ position (Hess and Fila, 2016a, 2016b).

Among these, the four-stage design empathy framework proposed by Kouprie and Visser (2009) is widely regarded as one of the most extensively applied and influential models in design research (Kouprie and Visser, 2009). (1) Discovery. The design process begins with initial contact between designers and users. Through communication with users or analysis of user research materials, designers develop a motivation to explore users’ contexts and experiences. (2) Immersion. Designers actively enter the users’ modes of thinking and attempt to observe from users’ perspectives. Kouprie and Visser (2009) argued that this stage plays a pivotal role in the formation of empathy and is essential for enabling designers to develop a deeper understanding of others’ circumstances (Kouprie and Visser, 2009). (3) Connection. Designers build connections with users by drawing on their own experiences and memories. At the affective level, they attune to users’ situations, and at the cognitive level, they make sense of the logic underlying users’ behaviours and needs, thereby achieving a reflective understanding in which affective and cognitive processes operate in parallel. Related work further suggests that effective learning experiences should encompass multidimensional engagement, including behavioral, cognitive, affective, and social involvement (Xie, 2021). (4) Detachment. Designers temporarily step back from the affective connection, return to the role of designer, and translate the understanding developed in earlier stages into design insights that support ideation and design decision making. In doing so, the process shifts toward problem solving and solution generation.

The four-stage framework described above provides a direct basis for constructing the empathy-driven instructional approach in the present study. For example, Tracey and Baaki (2022) applied this framework in an authentic design project course to guide students through the stages of engaging with learners, entering learners’ contexts, establishing affective and cognitive connections, and eventually returning to the designer role to produce design deliverables (Tracey and Baaki, 2022). Chen G. et al. (2025); Chen J. et al. (2025) also used this framework to organize user-understanding activities in design projects, thereby promoting students’ reflection on and revision of their assumptions about user needs (Chen G. et al., 2025; Chen J. et al., 2025). Shi et al. (2025) further showed that role simulation and experiential prototyping can be understood as concrete applications of the four-stage empathy process (Shi et al., 2025).

This framework is also applicable to CPD education. CPD courses inherently involve sequential processes such as cultural information identification, meaning translation, and solution implementation, which are highly consistent with the design process emphasized in this model. Therefore, the present study adopted the four-stage empathy framework to construct an instructional approach for CPD.

### 2.4 Design creativity

Creativity, as a core cross-disciplinary concept, has long been defined from multiple perspectives (Sarkar and Chakrabarti, 2011). Amabile (1983) emphasized its process orientation and defined creativity as the process of producing outcomes that are judged as creative within a given social context (Amabile, 1983). By contrast, Jagtap (2019) and Jung et al. (2010) adopted an ability-based perspective and conceptualized creativity as an individual’s capability to generate ideas or outcomes that are both novel and valuable within a specific domain (Jung et al., 2010; Jagtap, 2019).

As research has progressed, scholars have increasingly focused on creativity in design contexts (Habib et al., 2025). Huang et al. (2024) examined the role of AIGC interventions in product ideation instruction in enhancing students’ creativity (Huang et al., 2024). Chandrasekera et al. (2024) investigated the influence of artificial intelligence in the early design process on students’ design creativity (Chandrasekera et al., 2024).

Within the above research framework, design creativity needs to be observed and evaluated through concrete design activities and explicit outputs. In design practice, concept sketches constitute a primary medium for expressing and developing design ideas, playing a critical role in advancing problem solving, refining concepts, and developing creative ideas (Do et al., 2000; Chen G. et al., 2025; Chen J. et al., 2025; Liu et al., 2026). They also provide one of the most direct windows for examining design creativity (Chu et al., 2017; Lee and Ostwald, 2022; Huang et al., 2024).

In prior research, Shah et al. (2000), drawing on the principles of statistical design of experiments, proposed four independent and effective metrics for evaluating concept sketches (Shah et al., 2000). Subsequent studies further elaborated these criteria (Shah et al., 2003; Cropley, 2006; Runco and Acar, 2012; Dumas et al., 2016; Daikoku et al., 2021). Fluency refers to the number of design solutions generated within a fixed time, reflecting the speed and breadth with which participants externalize ideas (Shah et al., 2003; Dong et al., 2023). Variety captures the diversity of distinct concepts produced in a design task, reflecting differences among creative concepts (Shah et al., 2003; Ritter and Ferguson, 2017).

Novelty is a key dimension for assessing the uniqueness and breakthrough character of solutions (Dumas et al., 2016; Chandrasekera et al., 2024; Li et al., 2024b). It reflects designers’ capacity for innovation in form, structure, function, and the reconstruction of cultural meanings, indicating the extent to which design outcomes depart from established paradigms and deliver new experiences (Shah et al., 2003; Hernandez et al., 2010). Quality concerns the feasibility and appropriateness of design outcomes from ideation to implementation (Sarkar and Chakrabarti, 2011). It encompasses technical viability, coherence between culture and function, and aesthetic performance (Shah et al., 2003; Dean et al., 2006; Verhaegen et al., 2013). This output-oriented sketch evaluation system provides structured and reusable criteria for assessing design creativity. Evaluations are typically conducted by experts or instructors to reflect the creative performance of learners’ design outputs.

Although dimensions such as fluency, variety, novelty, and quality are mainly derived from general research on design creativity, they remain highly applicable to studies of CPD education. Existing CPD research has also largely evaluated students’ creative performance from the perspective of design outcomes (Luo and Dong, 2017; Dong et al., 2023; Qin et al., 2025). However, these dimensions need to be further adapted and interpreted within the CPD context. CPD involves the identification of cultural elements, the extraction of meanings, and their translation into products. Therefore, novelty should not refer only to formal or functional uniqueness, but should also consider whether cultural elements have been reinterpreted, recombined, and creatively transformed. Similarly, quality should not be limited to technical feasibility and functional rationality, but should also examine whether cultural characteristics are meaningfully aligned with product functions, use contexts, and aesthetic expression (Qin et al., 2025).

Based on the multidimensional structure of design creativity described above and prior findings on the relationship between empathy and creative performance, this study further examines whether the empathy-driven instructional approach promotes different dimensions of students’ CPD creativity. Accordingly, the following hypotheses are proposed:

*H2a*: Compared with the traditional instructional approach, the empathy-driven instructional approach will be associated with a greater improvement in design fluency.

*H2b*: Compared with the traditional instructional approach, the empathy-driven instructional approach will be associated with a greater improvement in design variety.

*H2c*: Compared with the traditional instructional approach, the empathy-driven instructional approach will be associated with a greater improvement in design novelty.

*H2d*: Compared with the traditional instructional approach, the empathy-driven instructional approach will be associated with a greater improvement in design quality.

In addition, empathy may not only emerge as an outcome of instructional intervention but may also be associated with how students understand user needs, cultural contexts, and design problems. Based on the positive relationship between empathy and creative performance reported in prior research, this study further examines the associations between empathy and different dimensions of design creativity and proposes:

*H3a*: Higher empathy will be associated with higher design fluency.

*H3b*: Higher empathy will be associated with higher design variety.

*H3c*: Higher empathy will be associated with higher design novelty.

*H3d*: Higher empathy will be associated with higher design quality.

On this basis, exploratory mediation analyses are further conducted to examine whether empathy may constitute a potential indirect pathway through which the empathy-driven instructional approach influences different dimensions of design creativity.

## 3 Materials and methods

This study was situated in a CPD course for third-year undergraduate students majoring in industrial design. The course was organized around an authentic cultural and creative design task and functioned as a project-based design practice course. Its instructional focus was not merely on delivering knowledge about cultural artefacts and design, but on guiding students through the complete design process, from understanding cultural resources and translating cultural meanings to generating product solutions and submitting design portfolios. Within this course context, the present study systematically examined the pedagogical effects of the empathy-driven instructional approach in design education and its underlying mechanism.

By comparing the empathy-driven instructional approach with a traditional instructional approach, the study analyzes their effects on design students’ empathy and their performance in CPD outcomes, and further investigates the role of empathy in linking instructional approach to design outcomes. Overall, the study evaluates the effectiveness of empathy-driven instructional approach across three levels, namely instructional approach, psychological mechanism, and design outcomes.

### 3.1 Research framework and correlated variables

To address the research questions, this study developed a research framework grounded in design empathy models and relevant literature, as shown in Figure 1. Regarding variable specification, instructional approach was treated as the independent variable with two levels, namely a traditional instructional approach for CPD and an empathy-driven instructional approach for CPD. This variable was used to examine how different instructional approaches influence students’ empathy and design outcomes. For the mediator, this study treated empathy as a psychological learning variable that may link instructional approach to design outcome performance, with the aim of preliminarily examining its potential indirect association between the empathy-driven instructional approach and design creativity. As the dependent variable, the creative performance of CPD outcomes was assessed as the primary learning outcome, operationalized through four dimensions, namely fluency, variety, novelty, and quality.

**Figure 1:**
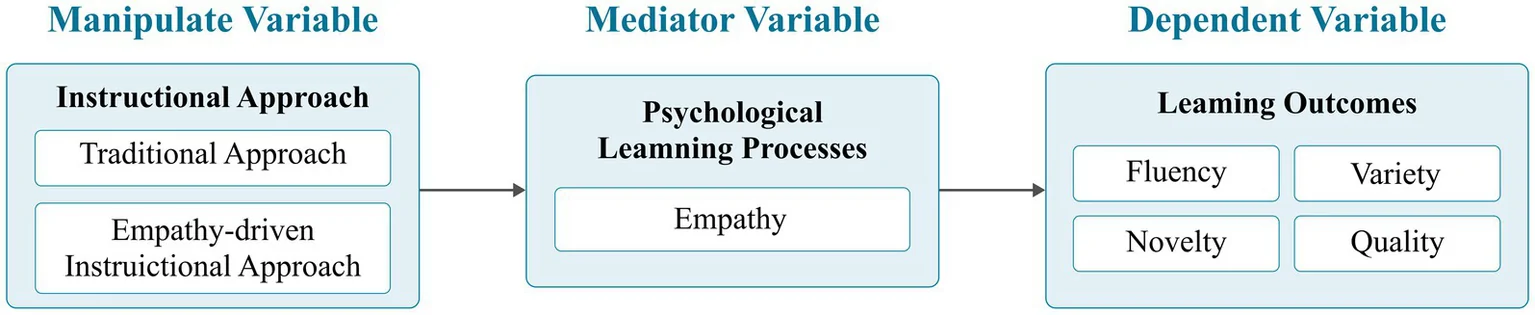
Research framework.

### 3.2 Participants

A total of 79 third-year undergraduate students majoring in industrial design at a university in central China were recruited from two intact classes of the CPD course. The control group comprised 39 students and the experimental group 40 students. Participants were 19–21 years old (*M* = 19.67, *SD* = 0.50); the mean age was 19.69 years (*SD* = 0.52) in the control group and 19.65 years (*SD* = 0.48) in the experimental group. The sample included 29 male students (36.7%) and 50 female students (63.3%). The control group included 15 males (38.5%) and 24 females (61.5%), whereas the experimental group included 14 males (35.0%) and 26 females (65.0%). No evident differences in age or sex composition were observed between the two groups.

Third-year students were selected because the course is offered in the third year as part of the actual undergraduate curriculum. By this stage, students had completed foundational courses in basic design methods, sketch representation, and product form design, and had acquired the basic design representation and concept-generation abilities required to complete CPD tasks. At the same time, their design practice experience was still developing, and they had not yet formed stable approaches to CPD. They therefore had the potential to respond to instructional intervention and demonstrate learning-related changes.

In terms of prior learning experience, the participants had been introduced to basic user research methods, such as needs analysis, user interviews, questionnaires, and user personas. However, this training had mainly served general product design problems, and their understanding of empathy was largely limited to the identification of user needs. Before the experiment, the participants had not received systematic empathic design training and generally lacked systematic knowledge of traditional culture and ancient artefacts. Therefore, this sample was suitable for examining the practical effects of the empathy-driven instructional approach in CPD education.

This study was conducted in an authentic course context and adopted a quasi-experimental design based on intact classes. In educational research, when individual random assignment is constrained by course organization and classroom management, the use of existing intact classes as experimental and control groups is common (Huang and Lyu, 2026; Wang et al., 2026). Because the two classes were comparable in professional background, year level, prerequisite courses, and class size, the original class structure was retained and the two intact classes were randomly assigned at the class level: one class was assigned to the empathy-driven instructional approach and the other to the traditional instructional approach. To further reduce the influence of non-target instructional factors, both classes completed the same design tasks over the same instructional period, followed identical assignment requirements and scoring criteria, and were taught by the same instructor according to a unified course schedule. Although instructional condition was randomly assigned at the class level, participants were not randomized individually; therefore, potential class-level influences such as classroom climate and peer interaction cannot be fully excluded. Accordingly, the study is treated as an intact-class quasi-experiment, and the findings are interpreted cautiously in light of this design.

### 3.3 Procedure

#### 3.3.1 Teaching implementation and course arrangement

This study was conducted within a 16-week CPD course. Two intact classes (Class A, *n* = 39; Class *B*, *n* = 40) were taught in parallel throughout the course by the same instructor following a standardized course structure. Drawing on staged instructional frameworks proposed by Dong et al. (2023) and Huang et al. (2024), the course was organized into two major design tasks, with Task 1 and Task 2 implemented under different instructional conditions (Dong et al., 2023; Huang et al., 2024). The schedule is shown in Table 1.

**Table 1:** Course schedule across 16 weeks.

Procedure	Weeks	Course activities
Pre-experiment	Weeks 1 to 2	Students learn the necessary theoretical background and methodological foundations of CPD.
Experimental treatment	Weeks 3 to 6	1. Students from two intact classes complete an open-ended CPD task without instructional intervention. 2. Empathy questionnaire, first measurement.
	Weeks 7 to 15	1. CPD task under different instructional approaches: Class A uses the traditional instructional approach and Class *B* uses the empathy-driven instructional approach. 2. Empathy questionnaire, second measurement in Week 12.
Submission	Week 16	Both classes submit the final design outcomes for Task 1 and Task 2 in the form of a portfolio.

During Weeks 1 to 2, all students received systematic instruction in the necessary theoretical background and methodological foundations of CPD. The content included basic concepts of CPD, identification of cultural elements, analysis of cultural meanings, and fundamental methods for translating cultural resources into product concepts.

During Weeks 3 to 6, both classes completed Task 1 in parallel. In this phase, all students worked under the same instructional condition. Each student selected one artefact from a provided collection as the inspiration source and independently completed a CPD task. No additional design guidance was provided for this task (Dong et al., 2023). After Task 1 was completed in Week 6, all participants were required to complete an empathy questionnaire.

During Weeks 7 to 15, the course moved into Task 2. Students in both groups selected a different artefact from the same collection that they had not used in Task 1 and completed a second CPD task. Class A served as the control group and followed the traditional instructional approach, whereas Class B served as the experimental group and followed the empathy-driven instructional approach. The specific configurations of the two instructional approaches are described in Section 3.3.2. At the same time, after the completion of the core instruction in Week 12, participants completed the second empathy questionnaire. To reduce the potential effects of memory, fatigue, and learning, the two measurements were conducted approximately 6 weeks apart, and the order of the questionnaire items was randomized.

In Week 16, all students submitted the final design outcomes for Task 1 and Task 2 in the form of a portfolio (Dong et al., 2023). The artefact stimuli were drawn from representative ancient collections curated by multiple authoritative museums. Most items were emblematic objects with distinctive cultural significance or high-level heritage value. The institutional credibility of the source museums and the authenticity of artefact preservation provided reliable visual stimuli for the experiment (Luo and Dong, 2017; Dong et al., 2023; Chen G. et al., 2025; Chen J. et al., 2025; Qin et al., 2025). To enhance comparability across stimuli and reduce potential confounding effects caused by material differences, this study selected four artefacts with identifiable formal structures and ornamental motifs and relatively similar levels of complexity and information content, thereby maintaining comparable functional attributes and visual load across stimuli, as shown in Figure 2.

**Figure 2:**
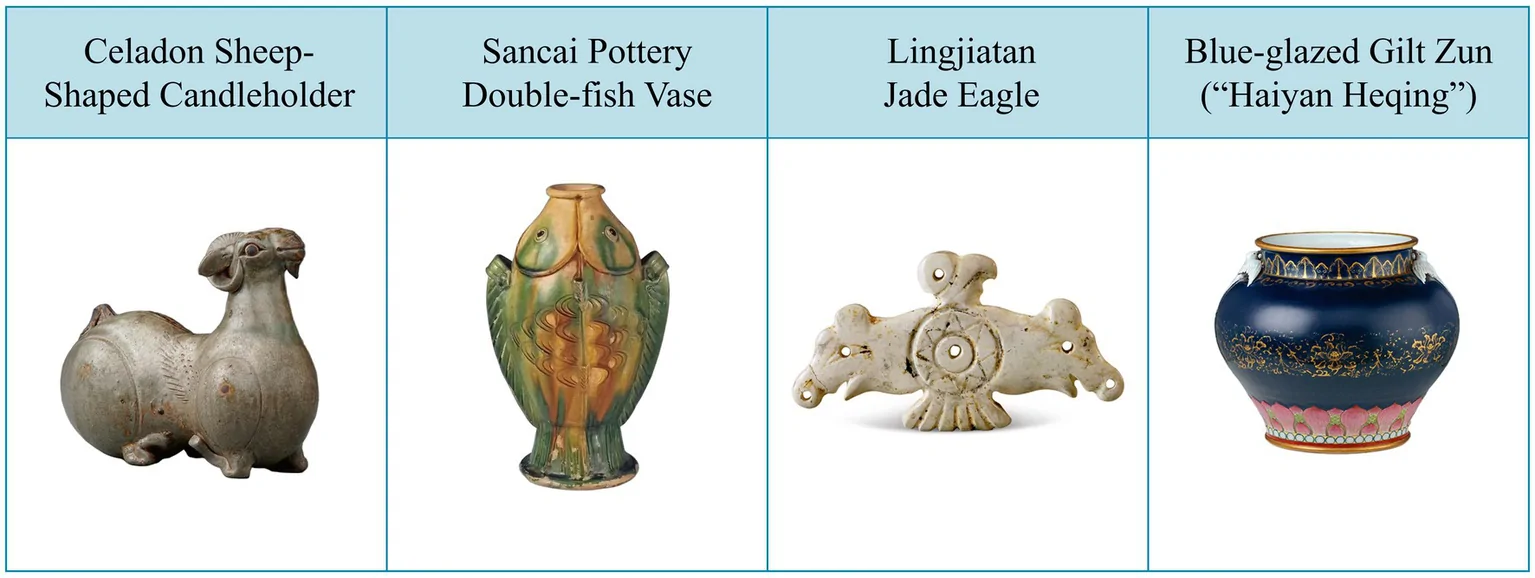
Artefact samples.

#### 3.3.2 Comparison between traditional and empathy-driven course frameworks

In the traditional instructional approach, the course framework was mainly based on the pedagogical approach proposed by Dong et al. (2023). To reduce the potential influence of differences in instructional input, both groups were required to complete information organization, staged analysis, design sketches, and solution explanations. The main difference lay in the cognitive pathway of the instructional scaffolding. The traditional instructional approach focused on identifying, extracting, and formally translating cultural elements, whereas the experimental group followed an empathy-driven instructional approach. This framework guided students from recognition of artefact forms toward understanding historical contexts and then toward analysing user behaviours, social relationships, cultural meanings, and contemporary cultural risks, supporting design translation with both cultural interpretability and contemporary relevance. Specifically, the framework comprised the following four stages:

(1) Discovery. Lin (2007) argued that the CPD process begins with the identification of cultural artefacts (Lin, 2007; Lin et al., 2007). Accordingly, the instructor provided both groups with the same basic artefact materials and defined information-source boundaries. Students in the experimental group first investigated the artefact name, historical period, original function, use environment, major formal features, and relevant cultural background and organized the information using a standardized template. This stage required an artefact background record to support subsequent reconstruction of historical contexts and user roles.
(2) Immersion. Based on the basic artefact information, students in the experimental group reconstructed the historical use context using a common set of guiding questions. They were required to determine who used the artefact, in what setting and for what purpose, and which behaviours and social relationships were involved in its use. On this basis, students constructed a prototypical role consistent with the historical use context, including the user’s identity or social role, relationship with the artefact, main purpose of use, and typical use setting. Students then mapped interactions between the role and the artefact according to the actual use process, recording key behavioural nodes, sequence, use environment, and relevant social relationships through a timeline, user-flow diagram, or sequential scenario sketches. This stage generated two required outputs:

1. A historical user profile describing the user’s identity, purpose of use, and relationship with the artefact; and.
2. A historical-context reconstruction showing the main behaviours, contexts, and social interactions involved in artefact use through a timeline, behavioural flow diagram, or scenario sketches.

(3) Connection. In this stage, structured learning tasks guided students to conduct active analysis using a standardized artefact analysis worksheet. Drawing on the dimensions of learning engagement proposed by Xie (2021), the worksheet included two core analytical components:

1. Cultural meaning analysis. Students identified the artefact’s principal form, motifs, materials, and functional features and further interpreted the symbolic meanings, identity expression, values, and sociocultural implications conveyed by these features. Particular attention was given to why these features appeared in a particular way and what they meant to historical users.
2. Cross-context empathic connection. Building on their understanding of the historical context, students compared the needs, emotional experiences, and cultural meanings of historical users with those of potential contemporary users. They analysed which meanings remained understandable and emotionally resonant and which might change because of differences in lifestyle, values, or use habits. This process was intended to establish connections among historical users, cultural meanings, and contemporary user experiences.

(4) Detachment. Students temporarily stepped out of the role perspective adopted in the preceding stage and returned to the designer’s standpoint to examine the contemporary applicability of cultural information. Using a standardized Empathy Reflection Notes template, students considered three questions:

1. whether the artefact’s original cultural meanings remained appropriate for expression in contemporary products;
2. whether its original functions and formal features remained reasonable and acceptable in contemporary use contexts; and.
3. whether cultural translation involved risks of misinterpretation, symbolic misuse, or displacement of cultural meaning.

Based on these judgements, students explicitly recorded which cultural information should be retained, transformed, or discarded and provided reasons for their decisions. The resulting reflections then served as the basis for design concept generation and refinement during Weeks 13–15. Based on this course design, the differences between the traditional instructional approach and the empathy-driven instructional approach are summarized in Table 2.

**Table 2:** Comparison between the traditional instructional approach and the empathy-driven instructional approach in CPD.

Weeks	Instructional stage	Common tasks	Control group	Empathy-driven group	Required outputs
7–8	Artefact understanding and contextual immersion	Acquire basic artefact information	Identify form, motifs, colour, and other cultural elements	Discovery + Immersion: background understanding, role construction, historical-context reconstruction	Information sheet; user profile; timeline/flow diagram/scenario sketches
9–11	Cultural meaning analysis	Analyse artefact functions and cultural features	Cultural element analysis sheet	Connection: cultural meaning interpretation and historical–contemporary user connection	Artefact context analysis worksheet
12	Contemporary applicability judgement	Evaluate appropriateness of cultural translation	Assess function, form, and cultural recognizability	Detachment: reflect on contemporary applicability, user acceptance, and cultural risks	Empathy reflection notes
13–15	Design translation and solution development	Concept generation, refinement, and explanation	Translate cultural elements into design	Translate empathic understanding into design	Concept sketches; final proposal; design rationale

### 3.4 Operational definition and measure of variables

#### 3.4.1 Scoring methods for multidimensional design creativity

Fluency was used to measure the number of valid design ideas generated by participants within the prescribed design period (Shah et al., 2003). In this study, a complete idea had to depict an identifiable product type, a basic function or use context, and a relatively complete core form in the sketch. Consecutive refinements of the same core concept were not counted repeatedly. Incomplete sketches, sketches with an unidentifiable design intention, and sketches that only changed colour, size, or local decoration without creating an independent design direction were not counted as new ideas. Each valid and independent idea received one point, and a participant’s fluency score for a task equalled the total number of complete and independent design ideas. In Figure 3, Participant P7 generated five design ideas using the Celadon Sheep-Shaped Candleholder as the source of inspiration; therefore, the fluency score was 5.

**Figure 3:**
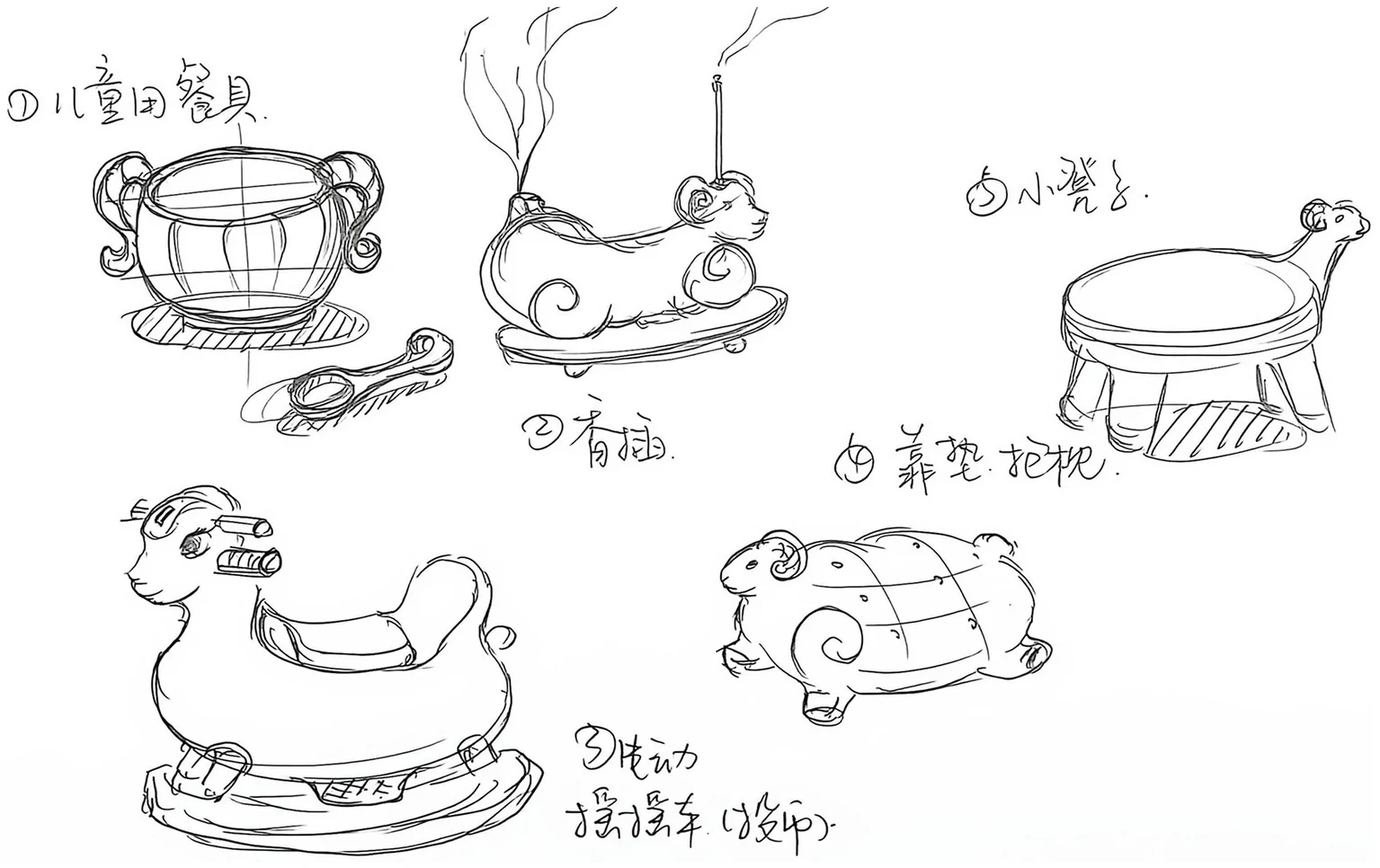
Sketches generated by participant P7 using the celadon sheep-shaped candleholder as the source of inspiration.

Variety was primarily assessed from three aspects. First, whether different concepts exhibited substantive differences in cultural-expression pathways, for example, developing ideas based on an artefact’s narrative meanings, symbolic significance, material characteristics, or use contexts. Second, whether functional ideation reflected different assumptions about product purpose, interaction modes, or use scenarios. Third, whether the concepts demonstrated clear differences in formal structure and technical principles (Qin et al., 2025). Following Huang et al. (2024), all concept sets were assessed using a four-level scoring scheme (1, 3, 6, 10), ranging from low to high (Huang et al., 2024). A score of 1 indicated that the ideas were largely homogeneous across these aspects and differed only in colour, size, or decorative detail; 3 indicated that the ideas largely followed the same core direction but showed a limited difference in one aspect; 6 indicated clearly distinct design categories in at least two aspects and relatively extensive exploration; and 10 indicated substantive differences in three or more aspects, forming multiple clear and distinguishable design pathways. The score was assigned to each participant’s complete set of ideas rather than by scoring and summing individual concepts. Participant P7’s five ideas (Figure 3) differed to some extent in function and use context, but several retained similar overall forms and structural expressions based mainly on the celadon sheep silhouette. Considering cultural expression, functional propositions, and structural form together, the variety score was 6.

Novelty refers to the unconventionality and degree of departure of the final design concept from common cultural products on the market. The evaluation focused on whether the design translated the artefact’s cultural meanings, such as symbolic significance and use contexts, in an unconventional manner; whether its functional proposition went beyond common cultural-product typologies; and whether concept generation yielded a distinctive and identifiable design proposal (Qin et al., 2025). Novelty was rated on a three-level scale of 3, 7, and 10 (Shah et al., 2003; Huang et al., 2024). A score of 3 indicated that the proposal was largely conventional across the three aspects and relied mainly on direct appropriation, surface decoration, or common functional combinations; 7 indicated a clear breakthrough in at least one key aspect while other aspects remained relatively conventional; and 10 indicated clear departures from conventional pathways in multiple key aspects, integrating cultural meaning, product function, and visual form into a highly distinctive original proposal. Participant P7’s proposal (Figure 4) combined the form of the celadon sheep with a multifunctional cushion, but similar products already existed in the market and the proposal did not sufficiently convey the artefact’s cultural meaning; therefore, the novelty score was 3.

**Figure 4:**
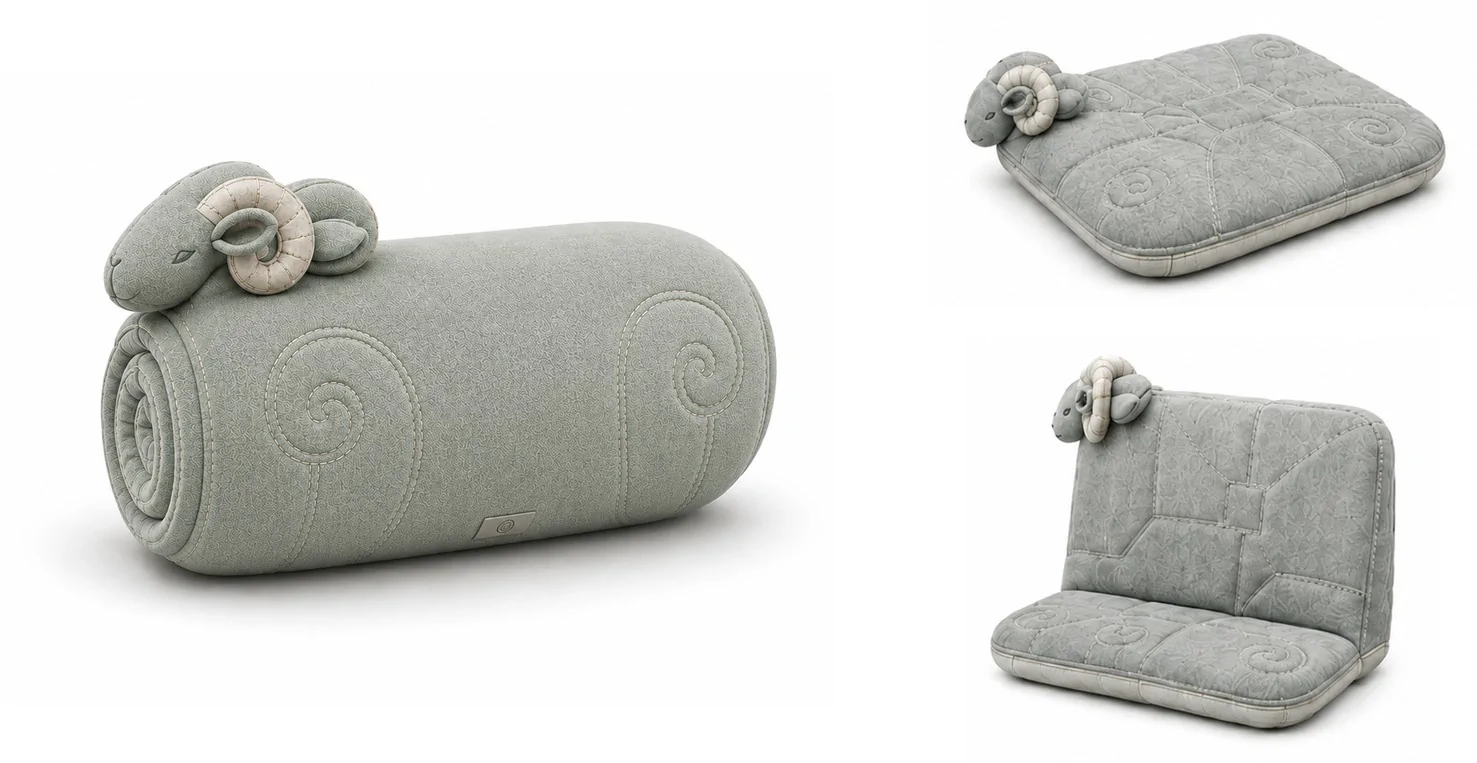
Rendering of Participant P7’s “Celadon Sheep Multifunctional Cushion”.

Quality refers to the overall performance of the final design proposal in technical and functional feasibility, appropriateness of cultural translation, fit with contemporary use contexts, and completeness of design expression (Qin et al., 2025). Technical and functional feasibility concerns the reasonableness of product structure, functional logic, and implementation; appropriateness of cultural translation concerns whether cultural elements are used accurately and appropriately and form a meaningful relationship with product function; contextual fit concerns whether the proposal aligns with contemporary users’ lifestyles and has clear cultural meaning and the potential to evoke emotional resonance; and completeness of design expression concerns the concept explanation, sketch communication, formal details, and overall maturity of the proposal. Quality was assessed using an overall four-level scale of 1, 3, 6, and 10. A score of 1 indicated fundamental deficiencies in feasibility, cultural expression, or completeness such that a viable product concept had not been formed; 3 indicated that the proposal broadly responded to the task but retained clear deficiencies in multiple aspects, such as functional logic, cultural translation, or design communication; 6 indicated that the proposal was generally feasible, that the relationship between cultural elements and product function was reasonably appropriate, and that the use context and expression were relatively complete, although some details still required improvement; and 10 indicated high technical and functional feasibility, accurate cultural translation fully integrated with contemporary use contexts, clear emotional meaning, strong aesthetic performance, and high overall maturity (Shah et al., 2003; Huang et al., 2024). The proposal shown in Figure 4 retains the formal and decorative features of the celadon sheep and integrates them relatively naturally into a contemporary home-use context, with clear functional logic and a relatively high level of overall completeness. However, its cultural elements are still expressed primarily through direct transfer of form and patterns, with limited further interpretation and translation of the deeper cultural meanings of the celadon sheep. The proposal therefore demonstrates generally good quality but still has room for improvement in the depth of cultural translation, resulting in a quality score of 6.

#### 3.4.2 An empathy scale in the context of cultural product design

This study used a six-item self-report empathy measure developed for the Cultural Product Design (CPD) context to assess students’ situated empathic engagement during specific design tasks rather than a stable personality trait.

The measure was primarily grounded in Drouet et al. (2024), particularly their operationalization of user perspective-taking, empathic understanding, and self–other distinction in design contexts. Harley et al. (2020), which demonstrates context-specific empathy measurement for particular target groups and learning situations, and Spreng et al. (2009), which defines general empathy in terms of understanding and attending to others’ feelings, provided complementary guidance for contextualizing the items (Spreng et al., 2009; Harley et al., 2020; Drouet et al., 2024).

Based on these theoretical and measurement foundations, the items were contextualized to reflect the distinctive CPD setting, which involves both historical cultural subjects and contemporary users. The six items do not represent independent empathy dimensions; rather, they jointly reflect a continuous process of situated empathic engagement in CPD: understanding historical users’ contexts and cultural meanings, recognizing differences between historical and contemporary user experiences, taking users’ perspectives, understanding their needs and feelings, and, after returning to the designer’s standpoint, using empathic understanding to judge the appropriateness and acceptability of cultural expression in contemporary contexts.

The initial item pool was developed through discussion within the research team and was then evaluated by two researchers with experience in design empathy research for face validity, content validity, and task relevance. Based on their suggestions, items weakly related to the CPD context or semantically redundant were removed, and overly behavioural or task-specific items were revised to more clearly capture empathic understanding, perspective-taking, and cultural interpretation.

The questionnaire was pilot-tested with 94 undergraduate design students whose characteristics were similar to those of the study participants. The pilot data indicated good measurement quality: Cronbach’s *α* = 0.86, KMO = 0.81, and Bartlett’s test of sphericity was significant (*χ^2^* = 168.27, *p* < 0.001). Confirmatory factor analysis indicated good fit for the one-factor model (CFI = 0.97, TLI = 0.95, RMSEA = 0.052, SRMR = 0.031), supporting the interpretation that the six items jointly reflected overall empathic engagement in the CPD context. The revised scale consisted of six positively scored items rated on a seven-point Likert scale, where 1 indicated “strongly disagree” and 7 indicated “strongly agree.” The arithmetic mean of the six items was used as each participant’s CPD-context empathy score, with higher scores indicating greater situated empathic engagement (Harley et al., 2020). The scale items are presented in Table 3.

**Table 3:** Empathy questionnaire items for the CPD context.

Item	Item content
Q1	I can understand the artefact’s uses, use constraints, and cultural meanings in historical life from the perspective of historical users.
Q2	I can recognize that the lifestyles, values, and needs of historical users may differ from those of contemporary users.
Q3	During CPD ideation, I can put myself in the position of historical users and imagine the processes, needs, and feelings they may have experienced when using the artefact.
Q4	I am attentive to and can understand the situations, needs, and feelings of historical users in their specific everyday lives.
Q5	I can understand the needs, experiences, and feelings that contemporary users may have when using cultural products from their perspective.
Q6	I can understand, from the perspective of contemporary users, how they may perceive and interpret the translated cultural expression, and whether such expression aligns with their ways of understanding and acceptance.

### 3.5 Statistical analysis

Descriptive statistics and Cronbach’s α were first used to examine the internal consistency of the empathy measure at the two measurement occasions. To examine the effects of instructional approach on empathy and design creativity, 2 (instructional approach: traditional vs. empathy-driven) × 2 (time: Task 1 vs. Task 2) mixed-design analyses of variance were conducted, with instructional approach as the between-participant factor and time as the within-participant factor. Significant group × time interactions were followed by simple-effects tests based on estimated marginal means, with Holm–Bonferroni correction for multiple comparisons.

The four design creativity dimensions were analysed separately. Inter-rater reliability of expert ratings was assessed using intraclass correlation coefficients. To explore the potential mediating role of empathy between instructional approach and design creativity, path analyses were conducted. Instructional approach was coded 0 = traditional and 1 = empathy-driven; T2 empathy was specified as the mediator; and T2 fluency, variety, novelty, and quality were analysed as separate outcomes. T1 empathy and the corresponding T1 creativity score were controlled in the outcome models. Indirect effects were estimated using 5,000 bias-corrected bootstrap samples to obtain 95% confidence intervals. Given that the instructional intervention was implemented at the intact-class level and each instructional condition corresponded to only one class, classroom-related factors and instructional conditions were partially confounded. Therefore, the present study adopted student-level analyses and interpreted the findings within the context of a quasi-experimental design involving intact classes.

## 4 Results

### 4.1 Analysis of students’ empathy

To address RQ1 and test H1, the internal consistency of the empathy scale was examined separately at Task 1 (T1) and Task 2 (T2). Cronbach’s *α* was 0.843 at T1 and 0.915 at T2, indicating good internal consistency at both measurement occasions.

Because the study included a within-participant factor of measurement occasion (T1 vs. T2) and a between-participant factor of instructional approach (empathy-driven vs. traditional), a 2 × 2 mixed-design analysis of variance (ANOVA) was conducted to examine changes in empathy. Levene’s tests indicated that the homogeneity-of-variance assumption was satisfied for the pretest and gain scores (all *p*’s > 0.05). Means, standard deviations, and 95% confidence intervals are reported in Table 4; the group-by-time pattern is shown in Figure 5; and the ANOVA results are reported in Table 5.

**Table 4:** Descriptive statistics by instructional approach and measurement occasion.

Outcome	Traditional T1	Traditional T2	Empathy-driven T1	Empathy-driven T2
Empathy	4.12 (0.58) [3.93, 4.31]	4.21 (0.60) [4.01, 4.40]	4.14 (0.71) [3.91, 4.36]	5.26 (0.66) [5.05, 5.47]
Fluency	7.65 (2.68) [6.78, 8.52]	9.47 (3.03) [8.49, 10.46]	7.64 (1.98) [7.00, 8.27]	9.44 (2.31) [8.70, 10.18]
Variety	6.37 (1.87) [5.77, 6.98]	7.18 (2.32) [6.43, 7.93]	6.26 (1.75) [5.70, 6.82]	7.46 (1.90) [6.86, 8.07]
Novelty	6.36 (1.60) [5.84, 6.88]	7.37 (2.30) [6.63, 8.12]	6.55 (1.58) [6.04, 7.06]	9.70 (0.85) [9.43, 9.97]
Quality	5.71 (1.73) [5.14, 6.27]	7.08 (2.50) [6.27, 7.89]	5.47 (1.94) [4.86, 6.09]	9.65 (1.10) [9.30, 10.00]

**Figure 5:**
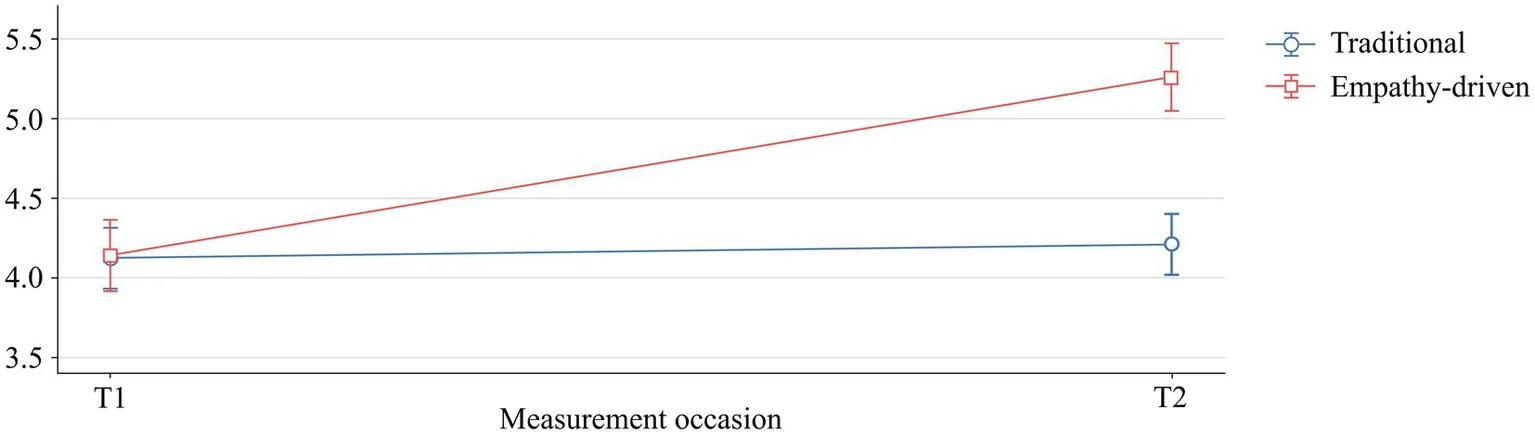
Mean empathy scores by instructional approach and measurement occasion. Error bars represent 95% confidence intervals.

**Table 5:** Mixed-design ANOVA results for empathy.

Effect	*F*(1, 77)	*p*	Partial *η*^2^
Group	17.43	<0.001	0.185
Time	85.36	<0.001	0.526
Group × time	61.53	<0.001	0.444

At T1, empathy was comparable in the traditional group (*M* = 4.12, *SD* = 0.58, 95% CI [3.93, 4.31]) and the empathy-driven group (*M* = 4.14, *SD* = 0.71, 95% CI [3.91, 4.36]). At T2, the mean increased to 5.26 in the empathy-driven group (*SD* = 0.66, 95% CI [5.05, 5.47]), compared with 4.21 in the traditional group (*SD* = 0.60, 95% CI [4.01, 4.40]). The mixed-design ANOVA revealed significant main effects of group, *F*(1, 77) = 17.43, *p* < 0.001, partial *η*^2^ = 0.185, and time, *F*(1, 77) = 85.36, *p* < 0.001, partial *η*^2^ = 0.526. More importantly, the group × time interaction was significant, *F*(1, 77) = 61.53, *p* < 0.001, partial *η*^2^ = 0.444, indicating that the two groups followed different trajectories over time.

Given the significant interaction, planned simple-effects comparisons based on estimated marginal means were conducted with Holm–Bonferroni correction. The groups did not differ at T1, *MD* = 0.018, 95% CI [−0.272, 0.308], *d* = 0.03, *p*_Holm_ = 0.903. At T2, however, the empathy-driven group scored significantly higher than the traditional group, *MD* = 1.053, 95% CI [0.769, 1.337], *d* = 1.66, *p*_Holm_ < 0.001. Within-group comparisons showed no significant change in the traditional group, *MD* = 0.085, 95% CI [−0.089, 0.260], *d_z_* = 0.16, *p*_Holm_ = 0.655, but a significant and large increase in the empathy-driven group, *MD* = 1.121, 95% CI [0.919, 1.322], *d_z_* = 1.78, *p*_Holm_ < 0.001. Thus, H1 was supported.

### 4.2 Analysis of design creativity

To address RQ2 and test H2a–H2d, two experts with more than 5 years of experience in cultural product design, design education, user experience, and empathy-driven design practice independently evaluated the portfolios submitted at the end of Week 16. To minimize evaluation bias, all portfolios were anonymized by removing students’ names, class information, and instructional condition and were presented using random identification numbers. The experts, who were blind to group membership, assessed each portfolio using common criteria for fluency, variety, novelty, and quality.

Inter-rater reliability was assessed using a two-way random-effects, absolute-agreement intraclass correlation coefficient based on the mean of two raters [ICC(2,2)]. As shown in Table 6, ICCs ranged from 0.857 to 0.980 across the four design creativity dimensions and the two measurement occasions, and all lower bounds of the 95% confidence intervals exceeded 0.75, indicating good-to-excellent inter-rater reliability. Accordingly, the mean of the two expert ratings was used in subsequent analyses.

**Table 6:** Inter-rater reliability of expert ratings across design creativity dimensions and measurement occasions.

Design creativity dimension	T1: ICC (2,2) [95% CI]	T2: ICC (2,2) [95% CI]
Fluency	0.969 [0.951, 0.980]	0.980 [0.968, 0.987]
Variety	0.884 [0.819, 0.926]	0.924 [0.881, 0.951]
Novelty	0.919 [0.874, 0.948]	0.932 [0.893, 0.956]
Quality	0.857 [0.777, 0.909]	0.926 [0.884, 0.953]

Mixed-design ANOVAs were conducted for each creativity dimension, with Holm–Bonferroni correction used to control the familywise error rate across follow-up comparisons. Levene’s tests indicated that the homogeneity-of-variance assumption was satisfied for the pretest and gain scores of all dimensions (all *p*’s > 0.05); standard *F* statistics were therefore used. Descriptive statistics are reported in Table 4, ANOVA results in Table 7, and the group-by-time patterns with 95% confidence intervals in Figure 6.

**Table 7:** Mixed-design ANOVA results for the design creativity dimensions.

Dimension	Time: *F* (1,77), *p*, partial *η*^2^	Group: *F* (1,77), *p*, partial *η*^2^	Time × group: *F* (1,77), *p*, partial *η*^2^
Fluency	168.70, <0.001, 0.687	0.00, 0.962, <0.001	0.01, 0.942, <0.001
Variety	28.76, <0.001, 0.272	0.05, 0.829, 0.001	1.09, 0.299, 0.014
Novelty	105.80, <0.001, 0.579	16.19, <0.001, 0.174	27.52, <0.001, 0.263
Quality	163.90, <0.001, 0.680	10.44, 0.002, 0.119	41.32, <0.001, 0.349

**Figure 6:**
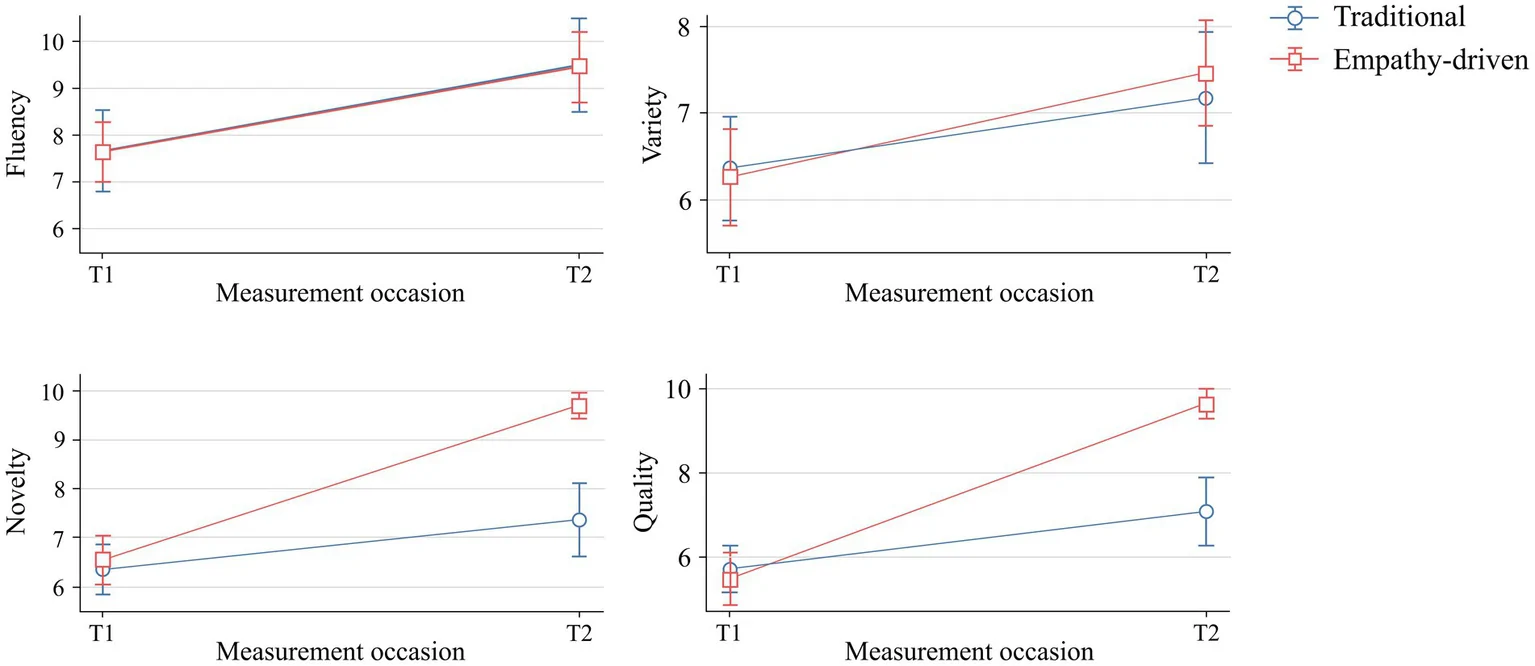
Mean design creativity scores by instructional approach and measurement occasion. Error bars represent 95% confidence intervals.

For fluency, both groups improved from T1 to T2 (traditional: 7.65 to 9.47; empathy-driven: 7.64 to 9.44), yielding a significant main effect of time, *F*(1, 77) = 168.70, *p* < 0.001, partial *η*^2^ = 0.687. Neither the main effect of group, *F*(1, 77) = 0.00, *p* = 0.962, partial *η*^2^ < 0.001, nor the group × time interaction, *F*(1, 77) = 0.01, *p* = 0.942, partial *η*^2^ < 0.001, was significant. Variety likewise showed a significant main effect of time, *F*(1, 77) = 28.76, *p* < 0.001, partial *η*^2^ = 0.272, but no significant main effect of group, *F*(1, 77) = 0.05, *p* = 0.829, partial *η*^2^ = 0.001, or group × time interaction, *F*(1, 77) = 1.09, *p* = 0.299, partial *η*^2^ = 0.014. Thus, both instructional approaches improved fluency and variety, but the empathy-driven instructional approach did not confer an additional advantage. H2a and H2b were not supported.

Novelty showed significant main effects of time, *F*(1, 77) = 105.80, *p* < 0.001, partial *η*^2^ = 0.579, and group, *F*(1, 77) = 16.19, *p* < 0.001, partial *η*^2^ = 0.174, as well as a significant group × time interaction, *F*(1, 77) = 27.52, *p* < 0.001, partial *η*^2^ = 0.263. The groups did not differ at T1, *MD* = 0.191, 95% CI [−0.522, 0.904], *d* = 0.12, *p*_Holm_ = 0.595, but the empathy-driven group scored significantly higher at T2, *MD* = 2.328, 95% CI [1.555, 3.101], *d* = 1.35, *p*_Holm_ < 0.001. Novelty increased by 1.013 points in the traditional group, 95% CI [0.270, 1.756], *d_z_* = 0.44, *p*_Holm_ = 0.018, and by 3.150 points in the empathy-driven group, 95% CI [2.779, 3.521], *d_z_* = 2.71, *p*_Holm_ < 0.001.

Quality also showed significant main effects of time, *F*(1, 77) = 163.90, *p* < 0.001, partial *η*^2^ = 0.680, and group, *F*(1, 77) = 10.44, *p* = 0.002, partial *η*^2^ = 0.119, together with a significant group × time interaction, *F*(1, 77) = 41.32, *p* < 0.001, partial *η*^2^ = 0.349. The groups did not differ at T1, *MD* = −0.230, 95% CI [−1.054, 0.594], *d* = −0.13, *p*_Holm_ = 0.580, whereas the empathy-driven group scored significantly higher at T2, *MD* = 2.573, 95% CI [1.713, 3.433], *d* = 1.34, *p*_Holm_ < 0.001. Quality increased by 1.372 points in the traditional group, 95% CI [0.735, 2.009], *d_z_* = 0.70, *p*_Holm_ < 0.001, and by 4.175 points in the empathy-driven group, 95% CI [3.564, 4.786], *d_z_* = 2.19, *p*_Holm_ < 0.001. Accordingly, H2c and H2d were supported, and H2 was partially supported overall.

Inspection of the final portfolios indicated that, in Task 1, before systematic instruction in cultural product design, students tended to directly appropriate the overall form or local patterns of the artefact; most designs remained at the level of formal reproduction. For example, some students directly applied the jade eagle form to a kite or keychain and made only simple adjustments to proportion and material, reflecting a form-transfer-oriented strategy (Figures 7a,b).

**Figure 7:**
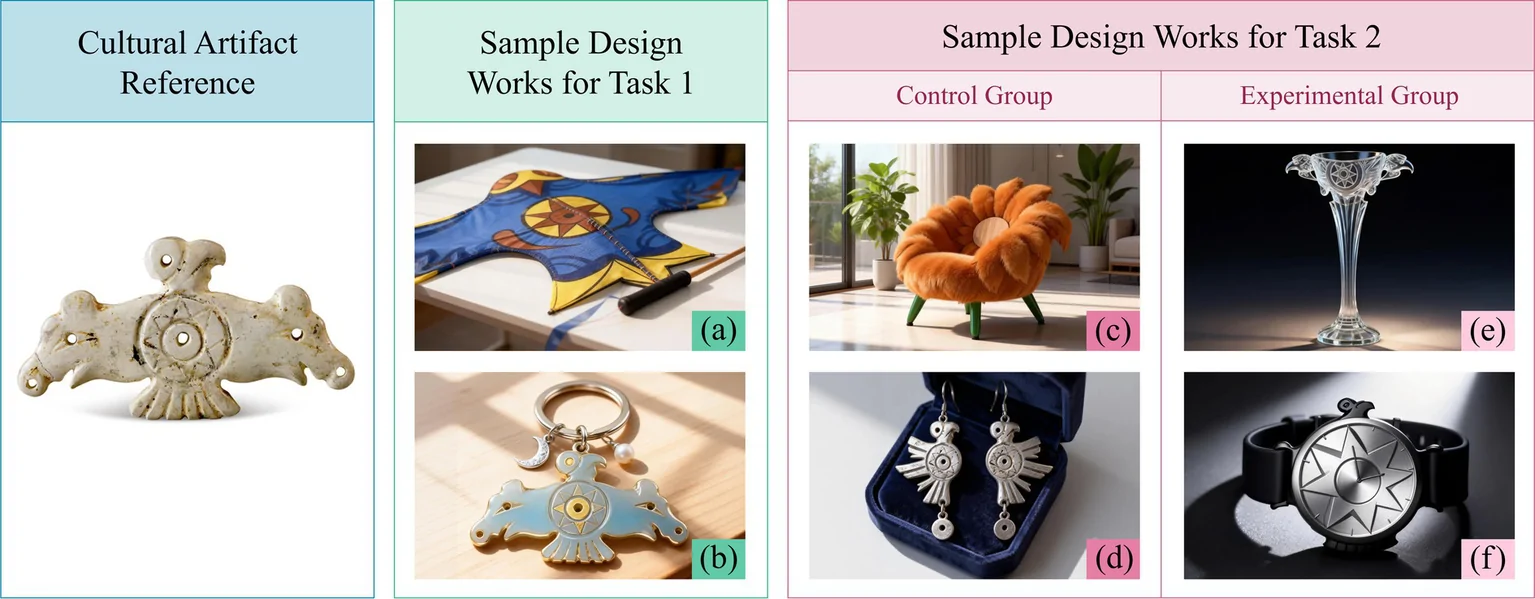
Representative examples of student design works across tasks and instructional groups.

After the instructional intervention in Task 2, students more deliberately extracted and translated artefact elements, although the depth and orientation of this translation differed by instructional approach. Works produced under the traditional instructional approach primarily focused on reorganizing formal language and visual features, such as geometrically transforming the central five-pointed-star motif into a sunflower-shaped chair or symmetrically adapting the eagle contour into earrings. These designs less frequently considered functional appropriateness in a specific use context or acceptance by contemporary users (Figures 7c,d). In contrast, students in the empathy-driven group more often began with the artefact’s original use attributes and cultural symbolism. For example, they translated the identity-related and social meanings of the jade eagle as a personal ornament into modern products such as trophies or watches, demonstrating integrated consideration of use contexts and user meanings (Figures 7e,f).

### 4.3 Exploratory mediation analysis of empathy

To address RQ3 and examine H3a–H3d, an exploratory mediation analysis was conducted using a path analysis framework. The independent variable was instructional approach (0 = traditional instruction, 1 = empathy-driven instruction), the mediator was T2 empathy score, and the dependent variables were T2 fluency, variety, novelty, and quality, respectively. The mediator model controlled for T1 empathy, while each outcome model additionally controlled for T1 empathy and the corresponding baseline creativity score. Indirect effects were estimated using 5,000 bias-corrected bootstrap samples. The complete path coefficients and 95% confidence intervals are presented in Table 8, and the significant mediation models are illustrated in Figure 8.

**Table 8:** Exploratory mediation analysis of empathy and design creativity outcomes.

Outcome	Total effect *c* B [95% CI], *p*	Path *a* B [95% CI], *p*	Path *b* B [95% CI], *p*	Direct effect *c*’ B [95% CI], *p*	Indirect effect *a* × *b* B [95% bootstrap CI]	Conclusion
Fluency	−0.020 [−0.582, 0.543] *p* = 0.945	1.043 [0.810, 1.276] *p* < 0.001	0.193 [−0.365, 0.750] *p* = 0.493	−0.221 [−1.031, 0.590] *p* = 0.589	0.201 [−0.233, 0.715]	Not significant
Variety	0.357 [−0.357, 1.070] *p* = 0.323	1.043 [0.810, 1.276] *p* < 0.001	0.192 [−0.514, 0.898] *p* = 0.590	0.157 [−0.870, 1.184] *p* = 0.762	0.200 [−0.560, 1.036]	Not significant
Novelty	2.232 [1.553, 2.912] *p* < 0.001	1.043 [0.810, 1.276] p < 0.001	1.073 [0.449, 1.697] *p* = 0.001	1.117 [0.209, 2.025] *p* = 0.017	1.119 [0.594, 1.735]	Consistent with partial mediation
Quality	2.670 [1.906, 3.433] *p* < 0.001	1.043 [0.810, 1.276] *p* < 0.001	1.320 [0.629, 2.011] *p* < 0.001	1.285 [0.276, 2.295] *p* = 0.013	1.377 [0.639, 2.280]	Consistent with partial mediation

**Figure 8:**
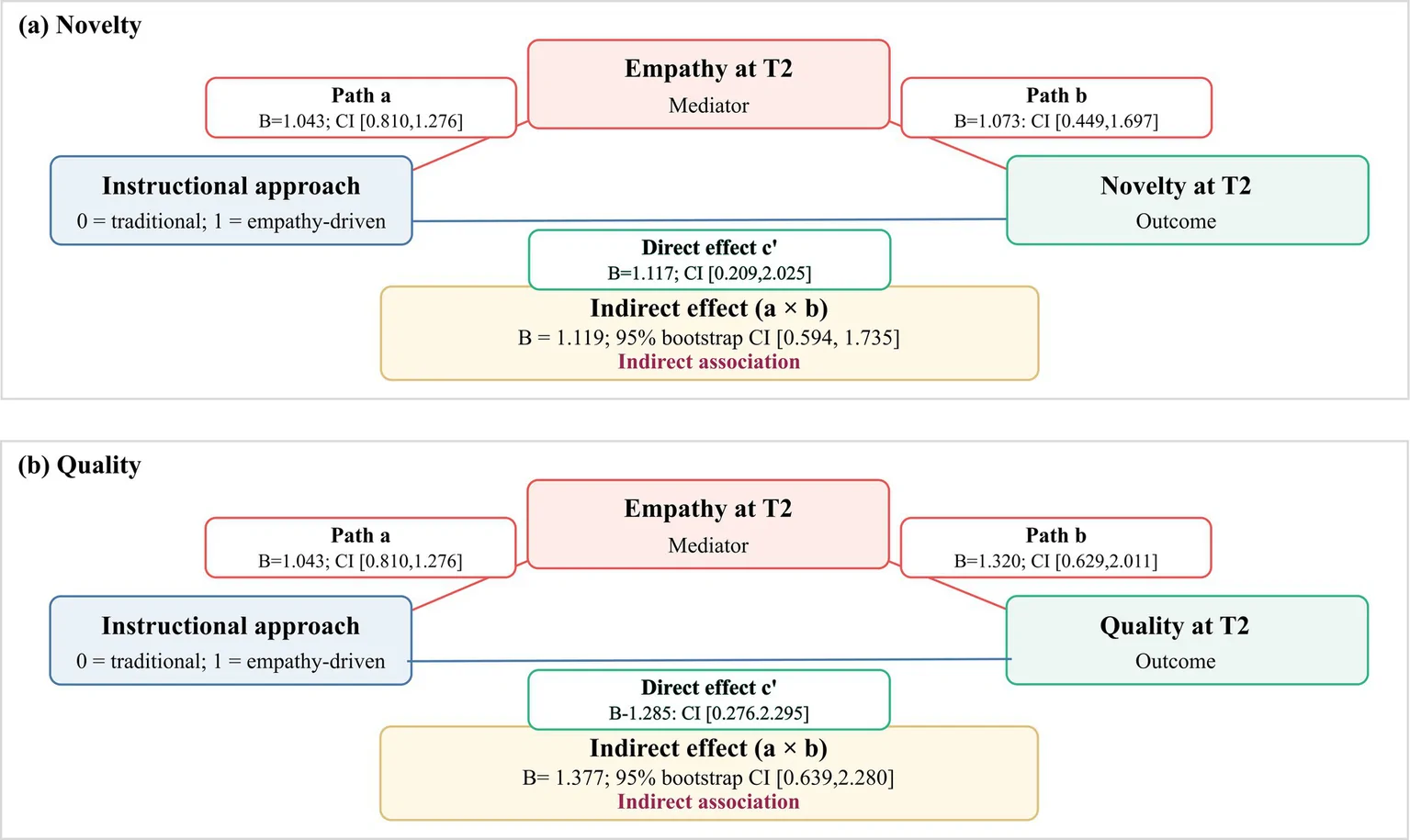
Exploratory mediation models for novelty and quality. Coefficients are unstandardized; indirect effects are reported with 95% bootstrap confidence intervals.

The instructional approach significantly and positively predicted T2 empathy, B = 1.043, SE = 0.117, 95% CI [0.810, 1.276], *p* < 0.001. This indicates that, after controlling for T1 empathy, students receiving empathy-driven instruction achieved an average increase of 1.043 scale points in T2 empathy compared with those receiving traditional instruction.

T2 empathy was not significantly associated with fluency, B = 0.193, SE = 0.280, 95% CI [−0.365, 0.750], *p* = 0.493, nor with variety, B = 0.192, SE = 0.354, 95% CI [−0.514, 0.898], *p* = 0.590. Correspondingly, the bootstrap confidence intervals for the indirect effects included zero (fluency: B = 0.201, 95% CI [−0.233, 0.715]; variety: B = 0.200, 95% CI [−0.560, 1.036]), indicating that H3a and H3b were not supported. In contrast, T2 empathy was positively associated with novelty, B = 1.073, SE = 0.313, 95% CI [0.449, 1.697], *p* = 0.001, and quality, B = 1.320, SE = 0.347, 95% CI [0.629, 2.011], *p* < 0.001. Therefore, H3c and H3d were supported.

After including T2 empathy in the model, the direct effect of instructional approach on novelty remained significant, B = 1.117, SE = 0.456, 95% CI [0.209, 2.025], *p* = 0.017. The direct effect on quality also remained significant, B = 1.285, SE = 0.507, 95% CI [0.276, 2.295], *p* = 0.013. Meanwhile, the indirect effect of instructional approach on novelty through empathy was 1.119, with a 95% bootstrap CI [0.594, 1.735]. The corresponding indirect effect on quality was 1.377, with a 95% bootstrap CI [0.639, 2.280]. Because neither confidence interval included zero and the direct effects remained significant, the results were consistent with a partial mediation pattern. Additional robustness analysis using a leave-one-out procedure confirmed that the main effects remained stable after removing individual participants (see Supplementary Material).

Taken together, H1 was supported. H2a and H2b were not supported, whereas H2c and H2d were supported; thus, H2 was partially supported. H3a and H3b were not supported, whereas H3c and H3d were supported; thus, H3 was partially supported. Table 9 summarizes the research questions, focal effects, and hypothesis-testing decisions.

**Table 9:** Summary of research questions, focal effects, and hypothesis-testing decisions.

Research question	Hypothesis	Measure/relationship	Conclusion
RQ1	H1: Compared with the traditional instructional approach, the empathy-driven instructional approach will be associated with a greater improvement in students’ empathy.	Empathy	Supported
RQ2	H2a: The empathy-driven instructional approach will be associated with a greater improvement in design fluency.	Fluency	Not supported
RQ2	H2b: The empathy-driven instructional approach will be associated with a greater improvement in design variety.	Variety	Not supported
RQ2	H2c: The empathy-driven instructional approach will be associated with a greater improvement in design novelty.	Novelty	Supported
RQ2	H2d: The empathy-driven instructional approach will be associated with a greater improvement in design quality.	Quality	Supported
RQ3	H3a: Higher empathy will be associated with higher design fluency.	Empathy → fluency	Not supported
RQ3	H3b: Higher empathy will be associated with higher design variety.	Empathy → variety	Not supported
RQ3	H3c: Higher empathy will be associated with higher design novelty.	Empathy → novelty	Supported
RQ3	H3d: Higher empathy will be associated with higher design quality.	Empathy → quality	Supported

## 5 Discussion

The findings indicate that, in an authentic CPD course, the empathy-driven instructional approach significantly enhanced students’ empathy and led to greater improvements in the novelty and quality of their design outcomes. By contrast, gains in fluency and variety primarily reflected general progress over the course rather than significant between-group differences. Exploratory mediation analysis further revealed a significant indirect association between changes in empathy and the advantages of the empathy-driven instructional approach in novelty and quality. Accordingly, Section 5.1 discusses how the empathy-driven instructional approach supports students’ empathy development; Section 5.2 examines possible reasons for its differentiated effects across dimensions of design creativity; Section 5.3 discusses the selective pattern of associations between empathy and different creativity dimensions; and Section 5.4 draws on the course implementation and findings to outline how empathy training may be embedded in CPD curricula and assessed pedagogically.

### 5.1 Empathy-driven instructional approach promotes students’ empathy development

The results indicate that, following the intervention based on the empathy-driven instructional approach, participants’ empathy in the CPD context increased significantly. This finding is consistent with prior research suggesting that empathy is not a fixed and stable individual trait, but can be effectively enhanced through targeted instructional design and learning activities (Chen G. et al., 2025; Chen J. et al., 2025; Shi et al., 2025).

From an instructional-process perspective, the observed change can be understood as the outcome of the continuous learning process created by the four-stage activities. During Discovery and Immersion, students first analysed artefact information, organized historical background materials, and reconstructed use contexts, gradually extending their attention from visible artefact forms to who used the artefact, in what social and cultural context, and for what purpose. This progression from object observation toward contextual understanding and role-based perspective-taking may have provided a basis for understanding historical users’ behaviours and experiences. This interpretation is consistent with evidence that narrative and contextual simulation can foster empathy (Tien et al., 2025).

In the connection stage, instruction encouraged students to understand artefacts and related cultural practices from the perspective of others, emphasizing an active entry into others’ experiences. Prior work indicates that deliberate training in perspective taking can substantially enhance individuals’ understanding of others’ standpoints and emotions, representing an important pathway for the development of empathy (Muñoz et al., 2025).

In the Detachment stage, students returned to the designer’s standpoint and reflected on the cultural understanding developed earlier and its applicability in contemporary contexts. Such reflective activities may help transform contextual experience into relatively stable cognitive judgement and may further support empathy (Badawy and Shaban, 2025). Importantly, the four stages were implemented as an integrated instructional framework rather than manipulated or compared independently. The present findings therefore support an association between the staged empathy framework as a whole and students’ empathy development but do not establish that any single stage is decisive.

### 5.2 Differential effects of the empathy-driven instructional approach across dimensions of design creativity

The findings of this study indicate that the empathy-driven instructional approach demonstrated significant advantages in novelty and quality, whereas its effects on fluency and variety were primarily reflected as overall time-related improvements. This pattern further suggests that the instructional approach does not operate by increasing the number or categories of ideas. Instead, it enhances the depth and effectiveness of design concepts by shaping how students understand the problem and define needs.

From a mechanistic perspective, the empathy-driven instructional approach facilitated other-oriented perspective shifting, encouraging students to interpret the design problem from the standpoint of the artefact and potential users. Prior research has shown that shifting between perspectives can help designers move beyond an egocentric stance and is considered an important psychological basis for creative thinking (Lamm et al., 2007), which can subsequently contribute to improvements in design quality (Hoever et al., 2012; Hu et al., 2021).

In addition, the reflective activities in the Detachment stage required students to reconsider the applicability of cultural meanings, functions, and forms within contemporary contexts (Seidel and Fixson, 2013). Previous research indicates that reflection can improve students’ creativity (Dumas et al., 2021), and that more accurate need definition is positively associated with design novelty (Adams et al., 2003). These reflective activities may therefore have encouraged students to judge more carefully how cultural information should enter contemporary products. By contrast, fluency and variety primarily depend on the frequency and breadth of idea generation. Such capacities may be more closely related to design-thinking training (Xia et al., 2021) and may not depend strongly on deep empathic processing (Yang and Yang, 2016).

### 5.3 Differential effects of empathy on different dimensions of design creativity

The present study further indicates that empathy did not show the same pattern of association across all dimensions of design creativity. Exploratory mediation analyses revealed significant indirect associations between instructional approach and novelty and quality through empathy, whereas the indirect effects for fluency and variety were not significant. This pattern suggests that empathy may be less closely associated with increases in idea quantity or categorical breadth and more closely associated with novelty, completeness, and the articulation of value in design proposals. This interpretation is consistent with previous research linking higher empathy to improved design quality and novelty (Yang and Yang, 2016; Demetriou and Nicholl, 2022).

A possible explanation is that empathy may shape students’ design outcomes by directing greater attention to user needs, contextual problems, and the interpretation of cultural meanings. Previous studies have indicated that empathy is closely related to intrinsic motivation. Individuals with higher empathy tend to proactively understand and alleviate others’ problems and therefore invest more cognitive and affective resources during the design process, which can contribute to design proposals with greater depth and completeness (Elsbach and Hargadon, 2006; Grant and Berry, 2011; Li et al., 2024a). In the present context, this may have encouraged students to consider not only which cultural forms to use, but also why particular cultural information should be used, what it means to users, and how it can be connected with contemporary product contexts, thereby supporting more targeted design judgements.

### 5.4 Pedagogical implications for CPD education

The empathy-driven instructional approach significantly enhanced students’ empathy in the CPD context, suggesting that staged empathy activities can function as pedagogical scaffolds within CPD courses. In the present implementation, Discovery can be supported by organizing artefact information and visual materials to establish basic understanding of historical period, function, and cultural background; Immersion can use historical-user role construction, reconstruction of use contexts, and scenario sketches to prompt students to consider who used the artefact, why, and how; Connection can use structured artefact-analysis worksheets to link formal features, functions, behaviours, social relationships, and cultural meanings; and Detachment can use reflection notes to prompt students to reconsider which cultural meanings should be retained, transformed, or reinterpreted. Together, the four stages can therefore be organized as a continuous sequence of cultural recognition, contextual understanding, meaning connection, and design judgement.

The findings further show that the advantages of the empathy-driven instructional approach were concentrated in novelty and quality, and portfolio analysis indicated that students in the experimental group were more likely to translate artefacts by considering original functions, cultural meanings, and contemporary use contexts. On this basis, activities within the empathy-driven instructional approach may be positioned before concept generation to deepen cultural understanding and design framing. For example, before sketching, students could complete a cultural-translation checkpoint requiring them to identify the artefact’s core cultural meanings, their relationship with the original use context, their applicability to contemporary life, and the product function or use scenario through which they will be translated. Such a checkpoint may encourage students to establish a connection between cultural meaning and design decision making before extracting visible forms or motifs, thereby reducing reliance on superficial formal translation. Because the present study found no additional advantage in fluency or variety, structured analysis can be relaxed once concept generation begins so that students retain sufficient space for open-ended creative exploration.

For assessment, the significant indirect associations of empathy with novelty and quality suggest that CPD courses may benefit from attending to students’ empathic development throughout the design process. In practice, a contextualized empathy measure can be combined with user-role and context analyses, artefact-analysis worksheets, reflection notes, and final design outcomes to support staged assessment. Early assessment can examine whether students understand historical users and cultural contexts; intermediate assessment can examine whether they connect cultural meanings, use behaviours, and product functions; and later assessment can examine whether these understandings are translated into novel, culturally appropriate, and well-developed design proposals. This approach extends assessment beyond final outcomes to sustained observation of cultural understanding, design judgement, and design translation.

## 6 Limitations

Although this study provides empirical evidence for the effectiveness of the empathy-driven instructional approach in cultural product design education, several limitations should be acknowledged. First, empathy was measured primarily through self-reported responses. Although the scale demonstrated good reliability and was specifically developed for the cultural product design context, self-reported measures may not fully capture students’ actual empathic engagement during the design process. In addition, the mediation analysis should be interpreted cautiously because empathy and creativity at T2 were measured at approximately the same time, and the relatively small sample size (N = 79) may limit the stability of indirect effect estimation. Future studies could incorporate qualitative evidence, such as systematic coding of students’ reflection notes, and adopt multiple time-point measurements with larger samples to further examine the potential mechanism underlying empathy development and design outcomes.

Second, the generalizability of the proposed CPD empathy framework requires further validation. The participants were recruited from a single university, and the cultural stimuli were limited to four visually recognizable historical artefacts with similar levels of complexity. Therefore, it remains unclear whether the framework can be equally applied to students from different educational contexts or to diverse cultural resources, including artefacts from different historical periods, material forms, and heritage categories. Future research should examine the applicability of the framework across broader cultural and educational settings.

Finally, this study adopted a quasi-experimental design using two intact classes. Although the two groups followed the same curriculum structure and were taught by the same instructor, classroom-level factors such as learning atmosphere and peer interaction could not be completely excluded. Future studies involving multiple classes or institutions could further strengthen causal inference through more rigorous experimental designs.

## 7 Conclusion

This study, situated in a university-level CPD course, empirically examined whether the empathy-driven instructional approach was associated with students’ empathy development and design creativity, providing empirical support for the central pedagogical question of how cultural understanding can be translated into creative design expression. The results indicate that, under the same design tasks and artefact cases, both instructional approaches supported the development of design creativity, whereas the empathy-driven instructional approach was associated with greater improvements in novelty and quality.

Exploratory mediation analyses identified significant indirect associations between the instructional approach and novelty and quality through empathy. This pattern suggests that empathy may be an important psychological variable for understanding the observed associations between the instructional approach and these outcomes.

Overall, the study provides empirical support for the value of the empathy-driven instructional approach in CPD education and offers a basis for further research on the interplay between affective and cognitive mechanisms in design education.

## Data Availability

The original contributions presented in the study are included in the article/supplementary material, further inquiries can be directed to the corresponding author.
