# Supplementary material for: Is empathy-driven teaching effective in cultural product design? An empirical study on students’ empathy and design creativity
Source: Front Psychol. 2026 Sep 29;17:1926260. doi: 10.3389/fpsyg.2026.1926260 (PMC13658138; doi:10.3389/fpsyg.2026.1926260)
Supplement: Supplementary file 1 [file Supplementary_file_1.DOCX]

# Robustness Analysis: Leave-One-Out Sensitivity Analysis

**Robustness analysis procedure.** To examine whether the main findings were driven by individual participants, a leave-one-out sensitivity analysis was conducted. Specifically, each participant was sequentially removed, and the main models were re-estimated across 79 iterations. The stability of the instructional effects on empathy and design creativity outcomes was evaluated based on whether the effects remained statistically significant after each iteration.

**Results.** The leave-one-out sensitivity analysis showed that the effects of empathy, novelty, and quality remained statistically significant across all 79 iterations. These results indicate that the observed effects were not driven by individual participants and demonstrate robustness to individual-level sample variation.

| Outcome | Original effect | Number of significant iterations | Total iterations | Conclusion |
| --- | --- | --- | --- | --- |
| Empathy | β = 1.043 | 79 | 79 | Robust |
| Novelty | β = 2.244 | 79 | 79 | Robust |
| Quality | β = 2.686 | 79 | 79 | Robust |
| Fluency | β = -0.020 | 0 | 79 | No significant effect |
| Flexibility | β = 0.366 | 0 | 79 | No significant effect |

**Interpretation.** The results confirm that the significant effects observed for empathy, novelty, and quality were stable after removing any single participant from the dataset. Therefore, the main conclusions regarding the effectiveness of empathy-driven instruction are unlikely to be attributable to individual-level outliers.
